# Ninjaflex vs Superflab: A comparison of dosimetric properties, conformity to the skin surface, Planning Target Volume coverage and positional reproducibility for external beam radiotherapy

**DOI:** 10.1002/acm2.13147

**Published:** 2021-03-10

**Authors:** Fiona M. Robertson, Megan B. Couper, Margaret Kinniburgh, Zoe Monteith, Gareth Hill, Sanka Andiappa Pillai, Douglas J. A. Adamson

**Affiliations:** ^1^ Radiotherapy Department Ninewells Hospital & Medical School NHS Tayside Dundee UK; ^2^ Medical Physics Department Ninewells Hospital & Medical School Dundee UK

**Keywords:** 3D printing, bolus, Ninjaflex, radiotherapy planning

## Abstract

**Background and purpose:**

When planning and delivering radiotherapy, ideally bolus should be in direct contact with the skin surface. Varying air gaps between the skin surface and bolus material can result in discrepancies between the intended and delivered dose. This study assessed a three‐dimensional (3D) printed flexible bolus to determine whether it could improve conformity to the skin surface, reduce air gaps, and improve planning target volume coverage, compared to a commercial bolus material, Superflab.

**Materials and methods:**

An anthropomorphic head phantom was CT scanned to generate photon and electron treatment plans using virtual bolus. Two 3D printing companies used the material Ninjaflex to print bolus for the head phantom, which we designated Ninjaflex1 and Ninjaflex2. The phantom was scanned a further 15 more times with the different bolus materials *in situ* allowing plan comparison of the virtual to physical bolus in terms of planning target volume coverage, dose at the prescription point, skin dose, and air gap volumes.

**Results:**

Superflab produced a larger volume and a greater number of air gaps compared to both Ninjaflex1 and Ninjaflex2, with the largest air gap volume of 12.02 cm^3^. Our study revealed that Ninjaflex1 produced the least variation from the virtual bolus clinical goal values for all modalities, while Superflab displayed the largest variances in conformity, positional accuracy, and clinical goal values. For PTV coverage Superflab produced significant percentage differences for the VMAT and Electron3 plans when compared to the virtual bolus plans. Superflab also generated a significant difference in prescription point dose for the 3D conformal plan.

**Conclusion:**

Compared to Superflab, both Ninjaflex materials improved conformity and reduced the variance between the virtual and physical bolus clinical goal values. Results illustrate that custom‐made Ninjaflex bolus could be useful clinically and may improve the accuracy of the delivered dose.

## 1 INTRODUCTION

Bolus materials are conventionally used in radiotherapy practice to alter the delivered dose to the skin surface and compensate for irregular patient contours. Naturally or synthetically developed materials have been used such as wet gauze, wax, and vinyl gels among others.^1^ Synthetic gel‐type commercial bolus for example, Superflab (Civco, Orange City, IA, USA), is in common use owing to its tissue equivalency (quoted density of 1.02 g/cm^3^) and being latex free. In clinical practice the positioning of bolus should be reproducible, and must maintain its shape and properties throughout the course of treatment.^2^ Direct contact of bolus with the surface of the skin is ideal as this is perceived to be more efficient by increasing the dose to superficial tissues and by improving dose uniformity. If bolus has not been applied closely to the skin surface then variations in air gaps during treatment may lead to a discrepancy between intended and delivered dose.^3^, ^4^ Kong and Holloway^5^ found that for electron beams the impact of air gaps was dependent on field size, beam energy, air gap size, and bolus thickness. For a 3 cm diameter circular field, 6 MeV beam, 20 mm air gap, and 15 mm bolus, both the maximum dose and surface dose were reduced by approximately 60%, and the depth of the dose maximum shifted by 3.5 mm. They recommended that air gaps should be avoided to improve the accuracy of treatment delivery. Butson et al.^6^ assessed the impact of air gaps for 6 MV beams using field sizes of 8 × 8 cm and 10 × 20 cm. They found that small air gaps (<10 mm) slightly decreased the surface and skin dose, but still allowed for at least 90% of the maximum dose being delivered to the skin regions.

Investigating alternative bolus materials for radiotherapy treatment may help to improve the accuracy of treatment delivery and patient outcomes. A promising tool that is enabling significant developments in radiotherapy is the three‐dimensional (3D) printer. 3D printing provides scope for printing out the exact patient surface contour, incorporating any unique indentations, and thereby accounting for individual differences.

A recent study by Park et al.^7^ investigated the use of patient‐specific breast bolus using 3D printed polylactic acid (PLA) bolus compared to their currently used Super‐Flex bolus. The 3D printed bolus was created from a computed tomography (CT) scan of a breast phantom. Treatment plans were generated to assess the effect of unwanted air gaps between the bolus and phantom’s surface on the dose distribution. The results showed that 3D printed solid bolus reduced variation in the daily setup and helped to overcome the dose discrepancy resulting from unwanted air gaps, leading to a more accurate treatment. It was concluded that 3D printed bolus could replace the currently used commercial boluses. Robar et al.^8^ analyzed the use of 3D printed PLA bolus for patients receiving chest wall radiotherapy compared with standard sheet bolus. Cone beam CT scanning was used to quantify the accuracy of fit with regards to air gaps between each type of bolus and the skin surface. For the sheet bolus, approximately 30% of all fractions involved air gaps of more than 5 mm, compared to 13% for the 3D printed bolus. They concluded that the accuracy of fit was improved significantly with the 3D printed bolus.

A literature review by Pugh et al.^9^ found that the improved conformity of 3D printed bolus could prove advantageous for volumetric modulated arc therapy (VMAT) and intensity‐modulated radiotherapy (IMRT) techniques as the presence of air gaps, small field sizes, and large beam obliquity can result in a reduction of 10% in the dose at the skin surface.

Recent studies have assessed the dosimetric properties and use of rigid, solid 3D printed plastics for boluses.^9^, ^10^, ^11^, ^12^ The disadvantage of these materials is their lack of flexibility. Ninjaflex, however, is a lightweight, flexible material that is rigid enough to hold its shape following printing.^13^ Robar et al.^11^ assessed the use of Ninjaflex as a bolus compared to standard sheet and 3D printed PLA bolus using a chest wall phantom. They found that both types of 3D printed boluses improved spatial conformity to the chest wall and that this improvement appeared to create a more uniform surface dose.

The aim of our study was to evaluate the suitability of 3D printed Ninjaflex bolus for external beam radiotherapy compared with the department’s standard bolus material, Superflab, by assessing dosimetric properties, conformity to the skin surface, planning target volume (PTV) coverage, and reproducibility of setup against a virtually created bolus produced within the treatment planning system (TPS).

## 2 MATERIALS AND METHODS

**Fig. 1:**
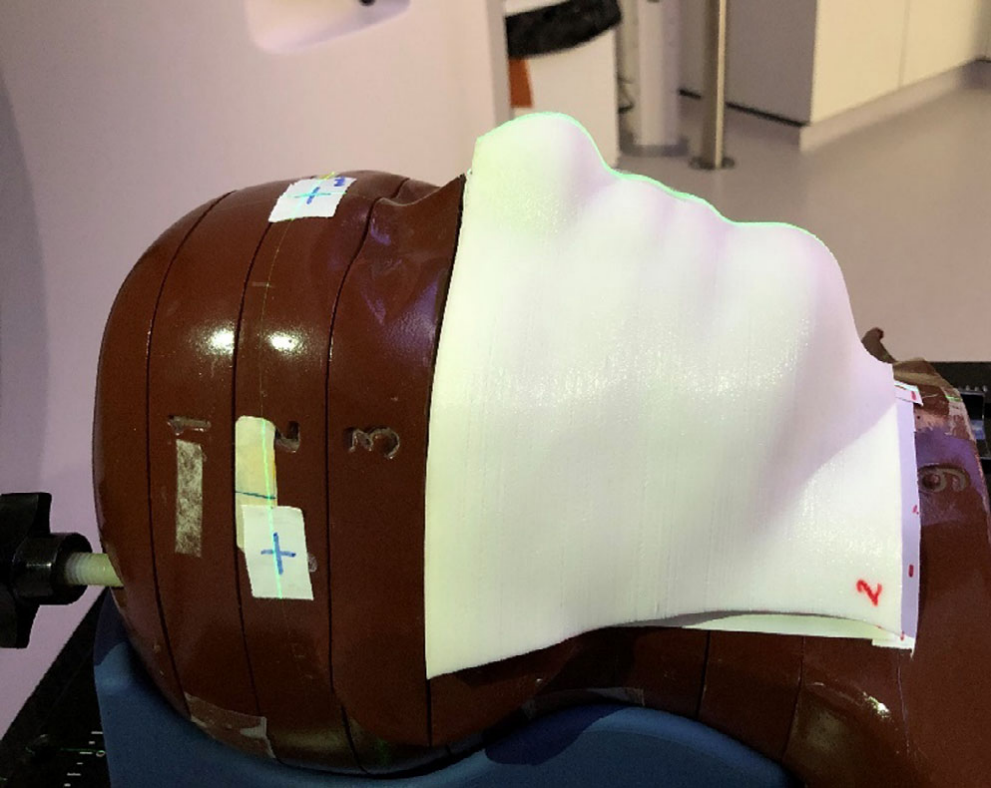
Positioning of three‐dimensional printed Ninjaflex bolus covering the right‐hand side of the head phantom to include the nose, lips, chin, and mandible.

The RANDO head phantom (The Phantom Laboratory, USA) was used for the evaluation as it was considered a difficult test surface to avoid air gaps between the bolus and skin surface owing to its contour.^6^ Internally the head phantom has the skeletal bone structure of a human skull; nasal, oral, and trachea air cavities; and teeth, all other tissue has a density of 1.00 g/cm^3^. An initial CT scan, using a Canon Aquilion Large Bore Multi Slice CT scanner (Canon Medical Systems Ltd, UK), was acquired and the image files were sent to two different 3D printing companies to generate the 3D printed Ninjaflex bolus. Ninjaflex is a thermoplastic polyurethane (TPU) material that is lightweight, promoted for its flexibility and longevity but is rigid enough to hold its shape following printing,^13^ suggesting that it could be an ideal material to create bespoke bolus. Company1 used a Ultimaker S5 to create Nijaflex1 while Company2 used a Lulzbot Taz 5 printer to create Ninjaflex2. The generated Ninjaflex bolus had a depth of 5 mm, and was designed to cover the right‐hand side of the head phantom including the nose, lips, chin, mandible, submaxillary triangle, extending posteriorly around the neck to the mastoid process and inferiorly to the thyroid cartilage (Figure 1). Each was requested to be 100% infill to minimize any density differences throughout the material.

The attenuation properties of the Ninjaflex material compared to Superflab were evaluated using ion chamber based measurements in solid water, performed with 0.5 × 10.0 × 10.0 cm and 1.0 × 10.0 × 10.0 cm bolus sheets for different photon and electron energies.

A CT scan of the phantom without bolus was carried out and a 5 mm depth virtual bolus (Bolus_Virt_) structure was created, with a density of 1.00 g/cm^3^, in the TPS RayStation (Software ver.5, RaySearch Laboratories, Stockholm, Sweden). From this CT dataset a PTV was delineated for both 3D conformal and VMAT plans and dose calculations were performed using a collapsed cone algorithm; three additional PTVs were produced for three electron plans which were calculated using a Monte Carlo algorithm, with 0.3 cm grid size and 300,000 histories. Each plan had a prescription of 20 Gy in 5 fractions. The 3D conformal plan had a PTV volume of 165.78 cm^3^ and consisted of five 6 MV fields incorporating wedges where required. The same PTV was used for the VMAT plan; two arcs rotated through gantry angles of 30° to 182°. Three electron plans (Electron1, Electron2, and Electron3) were created for three different PTVs situated under the bolus, with PTV volumes of 6.74, 13.15, and 1.23 cm^3^, using 9, 6, and 9 MeV, respectively.

Clinical goals currently implemented in the department were used: PTV coverage of at least 95% of prescription dose at 99% of the volume (D99); at most 105% of prescription dose to 5% of the volume (D5) and at most 107% of the prescription dose to 2% of the volume (D2). For the electron plans, the clinical goals used ensured that at least 90% of the prescription dose covered 99% of the PTV volume (D99). Maximum dose to the surface of the skin was set at 110% of the prescription dose for all plans.

To test the reliability of the physical bolus materials, the head phantom was scanned five times for each bolus, each time with different radiographers performing the setup. Using the RayStation plan evaluation the Bolus_Virt_ planned beam set dose was calculated on the separate CT datasets for comparison of the different physical bolus materials. We were, therefore, able to compare the Bolus_Virt_ to the physical bolus materials with regards to PTV coverage, dose at the prescription point, skin dose, and air gap volumes.

### 2.A Theory/calculation

The 3D printed Ninjaflex bolus was printed successfully by Company1 and Company2. Identified air gaps from the CT images, were contoured in the TPS to determine the volume of air between the bolus and skin surface. PTV coverage for dose minimum (D99) and maximum (D2) were recorded as well as dose at the prescription point. The data collected from the TPS were analyzed using the statistical software program SPSS Version 22. The Kruskal–Wallis test was used to determine if there were significant percentage differences (%_Diff_) between the Bolus_Virt_ and the different physical bolus materials with regards to the clinical goal values generated from each of the different plans. Spearson’s rho correlation coefficient was used to assess the relationship between air gaps and the clinical goal values.

## 3 RESULTS

The density of the phantom bolus was measured using the TPS and was found to be 0.96 g/cm^3^ for Ninjaflex1 and 1.02 g/cm^3^ for Ninjaflex2. The physical properties of each 5 and 10 mm square bolus sheet were measured to verify the density (Table 1). The 10 mm 3D printed Ninjaflex1 sheet was found to have a lower density than expected, which was apparent by the observed physical weight and feel of the bolus.

**Table 1:** Physical properties of the 5 and 10mm physical bolus to determine physical density.

Bolus	Length (mm)	Width (mm)	Depth (mm)	Volume (cm3)	Mass (g)	Physical Density (g/cm3)
Ninjaflex1_5mm	99.6	100.2	4.9	48.90	47.2	0.97
Ninjaflex1_10mm	99.6	100.5	10.6	105.90	80.5	0.76
Ninjaflex2_5mm	99.7	99.8	5.0	49.75	52.5	1.06
Ninjaflex2_10mm	100.0	99.6	9.9	98.62	104.0	1.06
Superflab_5mm	105.8	107.0	6.0	67.91	61.0	0.90
Superflab_10mm	103.4	93.8	9.0	87.26	94.0	1.08

**Fig. 2:**
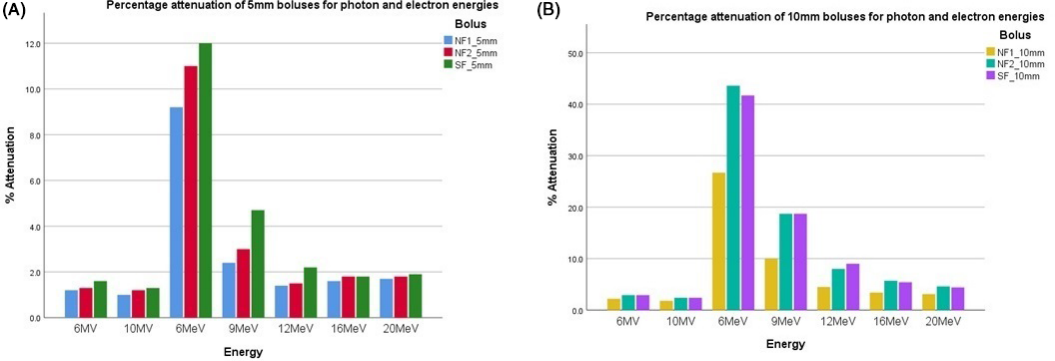
Attenuation characteristics for 5 and 10 mm bolus thicknesses for different photon and electron energies.

Figure 2 displays the percentage attenuation recorded for each bolus sheet and thickness. The attenuation characteristics for Ninjaflex2 and Superflab are similar. Ninjaflex1 displayed decreased attenuation for all energies tested, which was consistent with the lower density, particularly for the 10 mm thick sheet.

**Fig. 3:**
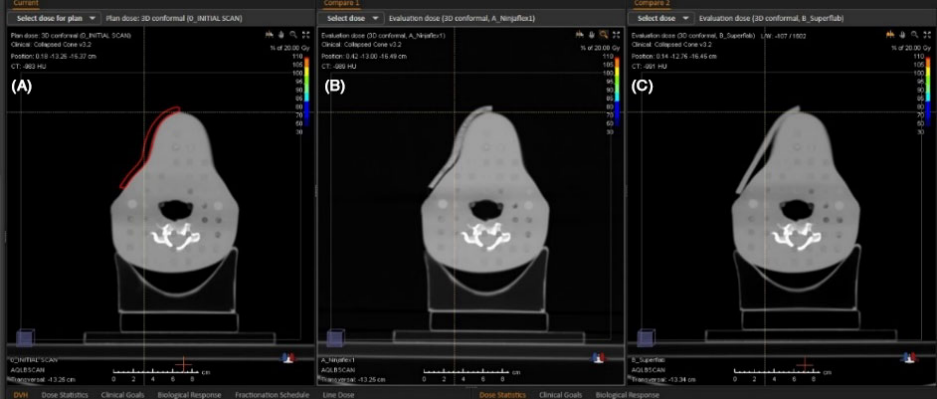
Conformity to skin surface from left to right Bolus_Virt_, Ninjaflex1 and Superflab.

Air gaps between the bolus and skin surface were analyzed on each CT and found to have statistically significant difference (*X^2^*(2) = 11.601, *P* < 0.05), with a mean rank of 12.6 for Superflab, 3.0 for Ninjaflex1 and 8.4 for Ninjaflex2. *Post hoc* tests were conducted to test pairwise comparisons. It was found that Superflab was significantly different from Ninjaflex1 (*P* < 0.01). Ninjaflex2 and Superflab and Ninjaflex1 and Ninjaflex2 were not significantly different. Superflab produced significantly more air gaps compared to Ninjaflex1, with the greatest volume of air being 12.02 g/cm^3^ and a significantly greater variation in reproducibility was observed. Figure 3 illustrates the difference between Bolus_Virt_, Ninjaflex1, and Superflab for the same CT slice.

The relationship between air gaps and D99, D2, prescription point dose, and skin dose on the 3D conformal and VMAT plans was statistically assessed. There was no correlation found between air gaps and D99 or D2 variables. There was a negative correlation between air gaps and prescription point variables [r = −0.704, n = 30, *P* < 0.01] with increases in air gaps correlated with a decrease in the prescription point %_Diff_. There was a positive correlation between air gaps and skin dose variable [r = 0.694, n = 30, *P* < 0.01]. Increases in air gaps correlated with an increase to the skin dose %_Diff_ compared to the Bolus_Virt_.

For the electron plans the relationship of air gaps showed no correlation between air gaps and D99; D2; prescription point; or skin dose variables. These results illustrate that, in this study, an increase in air gaps dose not correlate negatively or positively with D99, D2, prescription point or skin dose %_Diff_.

Table 2 shows the mean and standard deviations of the %_Diff_ virtual and physical bolus materials plans for D99, prescription point, D2 and air gaps.

**Table 2:** Mean and standard deviations for the clinical goal values of each treatment plan using the different bolus materials.

Plan	Bolus		D99 %_Diff_	Prescription Point %_Diff_	D2 %_Diff_	Air Gaps (cm^3^)
		*N*	*Mean (SD)*	*Mean (SD)*	*Mean (SD)*	*Mean (SD)*
3D conformal	Superflab	5	0.102 (0.036)	−0.130 (0.125)	−0.387 (0.132)	6.578 (3.825)
	Ninjaflex1	5	0.112 (0.043)	0.210 (0.055)	−0.341 (0.062)	0.114 (0.142)
	Ninjaflex2	5	0.061 (0.067)	−0.010 (0.074)	−0.379 (0.116)	2.174 (1.227)
VMAT	Superflab	5	−0.112 (0.022)	−0.101 (0.051)	0.000 (−0.790)	6.578 (3.825)
	Ninjaflex1	5	−0.040 (0.043)	−0.080 (0.045)	−0.060 (0.022)	0.114 (0.142)
	Ninjaflex2	5	−0.102 (0.036)	−0.111 (0.055)	−0.088 (0.027)	2.174 (1.227)
Electron1	Superflab	5	−0.907 (0.370)	1.062 (0.729)	−0.371 (0.680)	2.292 (2.907)
	Ninjaflex1	5	−1.204 (0.233)	−0.343 (0.800)	−0.649 (0.235)	0.000 (0.000)
	Ninjaflex2	5	−0.896 (0.336)	0.527 (0.634)	−0.330 (0.384)	1.319 (1.116)
Electron2	Superflab	5	−0.802 (0.163)	0.120 (0.800)	0.148 (0.367)	1.136 (0.703)
	Ninjaflex1	5	−1.440 (0.119)	0.460 (0.429)	−0.108 (0.235)	0.000 (0.000)
	Ninjaflex2	5	−0.945 (0.307)	0.140 (0.622)	−0.168 (0.205)	1.694 (1.270)
Electron3	Superflab	5	−4.090 (1.670)	−4.820 (1.727)	−5.100 (2.504)	4.700 (4.213)
	Ninjaflex1	5	−1.455 (1.028)	−2.340 (0.759)	−1.937 (0.394)	0.000 (0.000)
	Ninjaflex2	5	−1.753 (1.357)	−0.460 (0.463)	−0.326 (0.541)	0.616 (0.517)

Each plan was analyzed to determine whether there was a significant difference between the Bolus_Virt_ and physical bolus for each clinical goal. For the 3D conformal plan, a significant difference was found for prescription point %_Diff_ mean values (*X^2^*(2) = 10.732, *P* < 0.05), with a mean rank of 3.9 for Superflab, 13.00 for Ninjaflex1 and 7.1 for Ninjaflex2. D99 and D2 %_Diff_ were not significant.

*Post‐hoc* pairwise comparisons found that Ninjaflex1 was significantly different from Superflab (*P* < 0.01). Ninjaflex2 and Superflab and Ninjaflex1 and Ninjaflex2 were not significantly different. Ninjaflex1 increased the dose to the prescription point when compared to the Bolus_Virt_ and with the least variance compared to Ninjaflex2 and Superflab.

Analysis of the VMAT plan showed a significant difference of means for D99 %_Diff_ (*X^2^*(2) = 6.976, *P* < 0.05), with a mean rank of 5.5 for Superflab, 11.9 for Ninjaflex1 and 6.6 for Ninjaflex2. Prescription point and D2 %_Diff_ were not significant.

*Post‐hoc* pairwise comparisons found that Ninjaflex1 was significantly different from Superflab (*P* < 0.05). Ninjaflex2 and Superflab, and Ninjaflex1 and Ninjaflex2 were not significantly different. Although all physical bolus materials decreased the dose to D99 compared to the Bolus_Virt_, Ninjaflex1 produced the least %_Diff_ but with greater variance compared to Ninjaflex2 and Superflab.

Electron1 plan showed no significant difference in the means values for D99, prescription point, or D2 %_Diff_ for Superflab, Ninjaflex1, or Ninjaflex2.

Results for Electron2 plan showed that the D99 %_Diff_ of the different bolus materials is significant. D99 %_Diff_ (*X^2^*(2) = 8.933, *P* < 0.05), with a mean rank of 11.1 for Superflab, 3.2 for Ninjaflex1, and 9.7 for Ninjaflex2. Prescription point and D2 %_Diff_ were not significant.

*Post‐hoc* pairwise analyses found that Ninjalfex1 was significantly different from Superflab (*P* < 0.05). There was no significant difference between Superflab and Ninjaflex2 or Ninjaflex1 and Ninjaflex2. Ninjaflex1’s D99 %_Diff_ was significantly reduced compared to Superflab, however, Ninjaflex1 had less variance in results and no air gaps which lead us to consider whether the density difference was responsible for the D99 data.

**Fig. 4:**
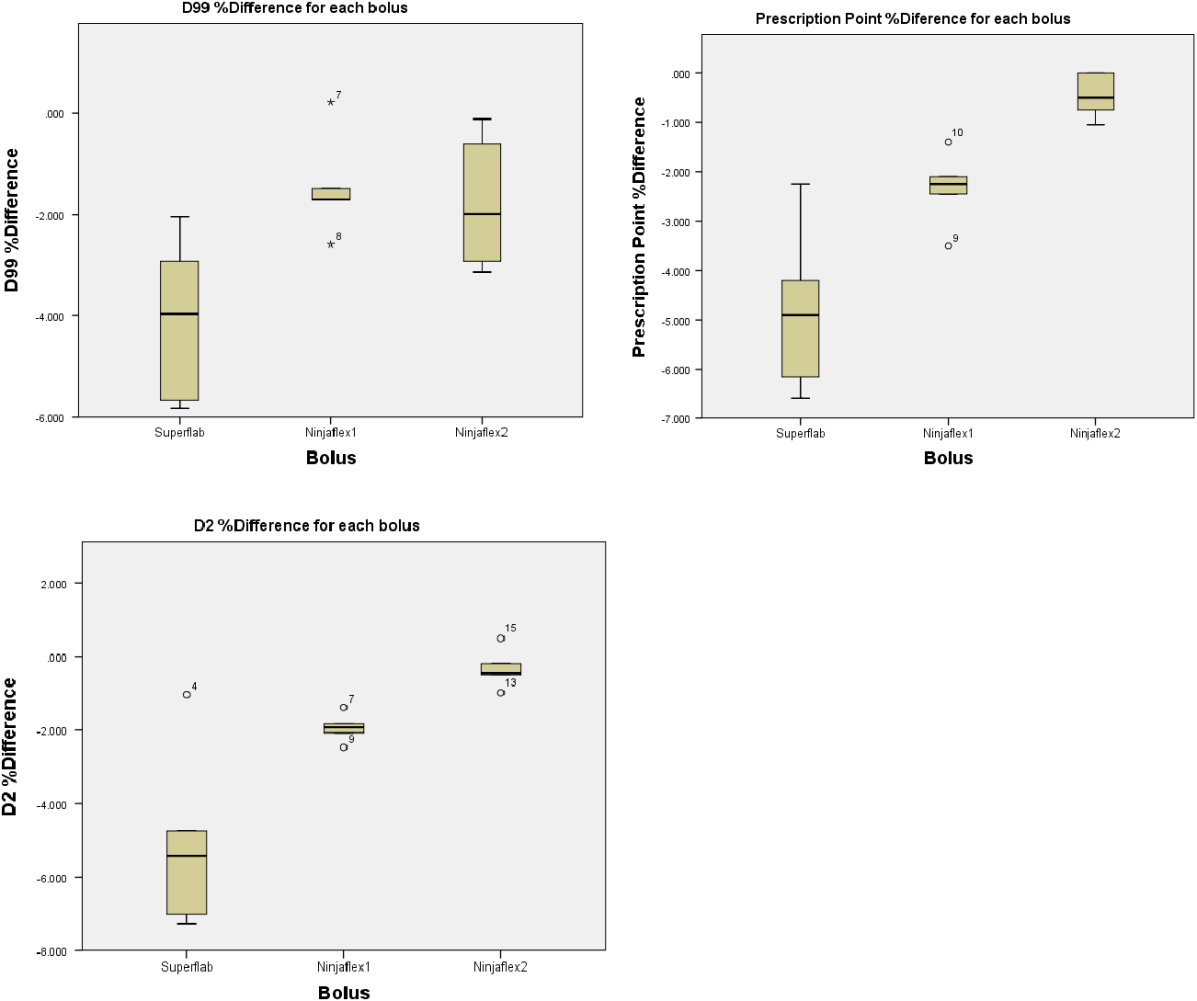
Summarizes the distribution in data between the different boluses and clinical goals depicting the median, first and third quartile, whiskers that extend no more than 1.5 times the interquartile (IQ) range and outliers whose values are between 1.5 and 3 times the IQ range. The numbered outliers refer to the Kruskal–Wallis test ranking value.

The Electron3 plan indicated a significant difference between D99, prescription point and D2 %_Diff_ across bolus groups. D99 %_Diff_ (*X^2^*(2) = 6.779, *P* < 0.05), with a mean rank of 3.9 for Superflab, 11.0 for Ninjaflex1 and 9.1 for Ninjaflex2. Prescription point %_Diff_ (*X^2^*(2) = 11.416, *P* < 0.01), with a mean rank of 3.5 for Superflab, 7.5 for Ninjaflex1 and 13.0 for Ninjaflex2. D2 %_Diff_ (*X^2^*(2) = 10.5, *P* < 0.01), with a mean rank of 4.0 for Superflab, 7.0 for Ninjaflex1 and 1.03 for Ninjaflex2. Figure 4 displays the distribution results for each clinical goal value.

D99 %_Diff_ *post‐hoc* pairwise analyses found that Ninjaflex1 was significantly different from Superflab (*P* < 0.05). There was no significant difference between Superflab and Ninjaflex2 or Ninjaflex1 and Ninjaflex2. D2 %_Diff_ *post hoc* analyses found that Superflab was significantly different from Ninjaflex2 (*P* < 0.01). There was no significant difference between Superflab and Ninjaflex1 and Ninjaflex1 and Ninjaflex2. Prescription point %_Diff_ *post‐hoc* analyses found a significant difference between Ninjaflex2 and Superflab (*P* < 0.01). There was no significant difference between Ninjaflex1 and Ninjaflex2 and Ninjaflex1 and Superflab. All physical boluses produced a reduction in clinical goal values compared to the Bolus_Virt_; Ninjaflex1’s D99 %_Diff_ demonstrated least reduction and with less variance in results. Ninjaflex2 showed least %_Diff_ and least variance for both prescription point %_Diff_ and D2 %_Diff_.

## 4 DISCUSSION

This study aimed to assess the feasibility of improving radiotherapy treatment by using a 3D printable flexible material for bolus. This was achieved by comparing clinical goal values of a virtual bolus created in the TPS to physical bolus materials, using different treatment modalities. Human factors in daily setup were also considered by changing the team of radiographers who applied the bolus and set the phantom up.

Ninjaflex1 resulted in significantly smaller air gaps, at most 0.32 cm^3^ of air, compared to Superflab demonstrating excellent conformity of the custom‐made 3D printed bolus to the phantom surface which is consistent with what other authors have shown in regards to conformity to skin surface.^4^, ^8^, ^14^ Data illustrates that Ninjaflex1 produced the least variance in the clinical goal values when compared to that achieved by the Bolus_Virt_ plan across all modalities. As predicted, Superflab displayed larger variances in positioning, conformity, and in achieving clinical goal values when compared to the Bolus_Virt_ plans. Furthermore, Ninjaflex2 showed no significant differences for any treatment modality or clinical goal values compared to the Bolus_Virt_ demonstrating that it too could be used as a more superior bolus material. Clinically, this would mean plans could be produced using Bolus_Virt_ and using the higher software version (Raystation ver. 7 or above) the bolus data can be exported to the 3D printer, resulting in improved conformity and positioning of the bolus. This was achieved by Baltz et al.^4^ when they created a patient‐specific 3D printed bolus scalp cap using RayStation scripting and the built‐in 3D print compatible function for exporting stereo lithography files to the 3D printer.

From previous research and accepted practice, air gaps are expected to result in significant differences from the planned dose for photon beams.^3^, ^4^ However, in this study they made minimal differences in PTV coverage, skin dose, and prescription point dose. In every case, no matter what bolus was used, all clinical goals were achieved. The greatest variance, for any parameter, was −0.57 %_Diff_ from the original planned dose. The VMAT plan illustrated a skin dose increase with an increase in air gaps. Electron therapy dose distributions, on the other hand, were significantly affected by the presence of air gaps and density differences, as expected. The mean dose to the D99 %_Diff_ decreased for all electron plans. Further analysis of the electron plans showed that when physical bolus was used to replicate Bolus_Virt_ the D99 coverage decreased by an average of 1.5% (*SD* = 1.23). The greatest variance was seen with Superflab, being −5.84 %_Diff_ from the planned dose using Bolus_Virt_. Ninjaflex1 had no visible air gaps for any of the electron plans and it was likely that the difference in D99 was owing to the known density difference between Bolus_Virt_ and Ninjaflex1.

It is acknowledged that the sample size for this study was small. Three different bolus materials were compared with Bolus_Virt_ for three different plan types. Physical bolus positioning was assessed for five CT scans; each acquired by a different set of radiographers to mimic the interindividual variation seen in day‐to‐treatment. The aim was to determine the feasibility of 3D printing and whether the 3D printed flexible material, Ninjaflex, could be used as a bolus clinically and whether it might be superior to our current standard, Superflab. We were able to assess whether setup adjustments and positioning of air gaps had an impact on the intended dose for different modalities, including VMAT. It was deemed appropriate to test the bolus materials five times to replicate a five‐fraction treatment and recording the %_Diff_ generated from the different modalities.

Health and Safety is a consideration in the health care environment, we found that both Ninjaflex’s appearance remained the same throughout the study, and microporous tape used for stability was removed easily leaving no residue. Unlike Superflab, each Ninjaflex bolus was individual so would not be able to be used with more than one patient. The Superflab, however, had microporous tape left on the surface and began to tear in some sections where more tape was used to avoid air gaps. There could therefore be infection control advantages to individualized 3D printed bolus.

Although the final 3D printed Ninjaflex from both companies were printed successfully, there were some initial errors were noted in the printed density. These errors resulted in air gaps within the bolus material which were rectified prior to starting the study. The size of bolus printed by Company2 differed to that of Company1. Adjustments were made to the Superflab and Ninjaflex1 so that all bolus materials were the same size for testing. Using the TPS to determine the area to be printed would help remove this potential error. To ensure quality assurance of the 3D print, it is recommended that calculating the expected mass and verifying that with the physical mass would confirm the correct corresponding Bolus_Virt_ density is used in the TPS. In‐house printing of bolus material may reduce the density variations due to the possibility of maintaining better quality control with the 3D printing task. Cost is also an important consideration: the final cost for the two head phantom prints was £1,173. Baltz et al.^4^ also reported similar costs of $2,381.50 when they used an external company to print out their scalp bolus. However, if the bolus was printed in‐house this cost would be reduced considerably; Canters et al.^15^ estimated costs of €28 for 3D printed bolus, although this is dependent on the size being printed.

## 5 CONCLUSIONS

We have tested the feasibility of using 3D printed Ninjaflex bolus as a substitute and improvement to our current standard, Superflab. We did this by assessing the dosimetric properties, conformity to the skin surface, PTV coverage, and reproducibility of setup against a virtually created bolus produced within the TPS. We found that Ninjaflex had excellent conformity and reduced the variance between the resultant virtual and physical bolus clinical goal values. The clinical user experience revealed that Ninjaflex was easier to position and setup compared with the Superflab as the Ninjaflex was ‘patient’ specific and conformed exactly to the surface of the phantom. There may also be infection control advantages in certain situations using a bolus that is patient specific. There are, however, cost considerations for single‐patient use bolus, although it may also be a preferred option for patients given the infection control benefits. Customized 3D printed Ninjaflex bolus could be used clinically and it may be that 3D printed bolus should be considered for certain types of treatment that appear to be more affected by the presence of air gaps, for example, electron therapy.

## CONFLICT OF INTEREST

Dr. Adamson reports other from NHS Tayside, during the conduct of the study; other from GSK, grants from Roche and Boehringer Ingelheim, other from Roche, outside the submitted work. Ms. Robertson reports grants from NHS Tayside, during the conduct of the study; other from GSK outside the submitted work.
